# Development and validation of a one year predictive model for secondary fractures in osteoporosis

**DOI:** 10.1371/journal.pone.0257246

**Published:** 2021-09-27

**Authors:** Setareh A. Williams, Susan L. Greenspan, Tim Bancroft, Benjamin J. Chastek, Yamei Wang, Richard J. Weiss, Nick Pyrih, Hily Nichols, Jane A. Cauley

**Affiliations:** 1 Health Economics and Outcomes Research, Radius Health, Inc, Boston, Massachusetts, United States of America; 2 Department of Medicine, University of Pittsburgh and University of Pittsburgh Medical Center, Pittsburgh, Pennsylvania, United States of America; 3 Department of Analytic Methods, Optum, Eden Prairie, Montana, United States of America; 4 Department of Immunology, Optum, Eden Prairie, Montana, United States of America; 5 Department of Biometrics, Radius Health, Inc, Boston, Massachusetts, United States of America; 6 Department of Global Medical Affairs, Radius Health, Inc, Boston, Massachusetts, United States of America; 7 Department of Advanced Analytics, Cobbs Creek Healthcare, Newtown Square, Pennsylvania, United States of America; 8 Department of Epidemiology, Graduate School of Public Health, University of Pittsburgh, Pittsburgh, Pennsylvania, United States of America; Medical College of Wisconsin, UNITED STATES

## Abstract

The number of osteoporosis-related fractures in the United States is no longer declining. Existing risk-based assessment tools focus on long-term risk. Payers and prescribers need additional tools to identify patients at risk for imminent fracture. We developed and validated a predictive model for secondary osteoporosis fractures in the year following an index fracture using administrative medical and pharmacy claims from the Optum Research Database and Symphony Health, PatientSource. Patients ≥50 years with a case-qualifying fracture identified using a validated claims-based algorithm were included. Logistic regression models were created with binary outcome of a second fracture versus no second fracture within a year of index fracture, with the goal of predicting second fracture occurrence. In the Optum Research Database, 197,104 patients were identified with a case-qualifying fracture (43% commercial, 57% Medicare Advantage). Using Symphony data, 1,852,818 met the inclusion/exclusion criteria. Average patient age was 70.09 (SD = 11.09) and 71.28 (SD = 14.24) years in the Optum Research Database and Symphony data, respectively. With the exception of history of falls (41.26% vs 18.74%) and opioid use (62.80% vs 46.78%), which were both higher in the Optum Research Database, the two populations were mostly comparable. A history of falls and steroid use, which were previously associated with increased fracture risk, continue to play an important role in secondary fractures. Conditions associated with bone health (liver disease), or those requiring medications that impact bone health (respiratory disease), and cardiovascular disease and stroke—which may share etiology or risk factors with osteoporosis fractures—were also predictors of imminent fractures. The model highlights the importance of assessment of patient characteristics beyond bone density, including patient comorbidities and concomitant medications associated with increased fall and fracture risk, in alignment with recently issued clinical guidelines for osteoporosis treatment.

## Introduction

The number of osteoporotic-related fractures in US is no longer declining [1,2]. Contributing factors include the demographic shift towards an aging population and a decrease in screening. In addition to limited primary prevention measures, secondary prevention to reduce the rate of subsequent fractures is also suboptimal. Most patients who incur a low trauma fracture do not undergo osteoporosis evaluation or initiate treatment [3] despite existing quality of care measures [4].

In the first year following initial fracture, patients have the highest risk of incurring a subsequent fracture. The rate of secondary fractures varies from 4–17% depending on initial fracture site and population characteristics [5,6], with the cumulative incidence increasing to 21% in the four years after the index fracture [7]. In a 10-year follow-up study, secondary fracture rates were 28% for patients with an initial hip fracture and 35–38% for those with non-hip fractures. The risk was highest in the first year after fracture (5–45%) and declined progressively during the 10-year follow-up [8]. There is an incremental cost for second fractures [5]. The total all-cause cost of care is significantly higher in the year following index fracture for those experiencing a second fracture compared to those without a prior fracture for both Medicare ($34,327 vs $20,790; *P* < .001) and commercial health plan enrollees ($39,501 vs $19,131; *P* < .001) [6].

Several risk assessment tools, including FRAX and GARVAN, predict the 5- or 10-year probability of fractures [9]. The identification of patients at high risk for subsequent fracture is important to payers, providers, and patients alike. Assessment of risk in the year immediately following index fracture, when the potential to reduce avoidable events and associated burden and cost of illness is greatest, is even more relevant.

The goal of the current study was to develop and validate a predictive model for secondary osteoporotic-related fractures in the year following an index fracture using administrative claims data.

## Material and methods

### Data source

The study included commercial and Medicare Advantage health plan members with evidence of a case-qualifying fracture between January 1, 2007, to May 31, 2017 (identification period) using the administrative claims data from the Optum Research Database (ORD). Optum has access to a proprietary research database with medical and pharmacy claims data (including linked enrollment data) from 1993 covering 59.5 million lives or approximately 10% of the US population.

Anonymized patient level data from Symphony Health, PatientSource were used for assessment of the model’s predictive performance in new individuals (external validation). Data are payer agnostic and provide access to individual-level healthcare claims for ˃280 million US-based commercial and Medicare Advantage enrollees. Unlike the ORD, the Symphony data includes enrollees from various US payers, but not from Optum. The study identification period was September 1, 2012 to October 31, 2018. The index date was defined as the first fracture claim during the identification period.

### Patient selection

Patients ≥50 years of age with a case-qualifying fracture during the identification period were included. Patients with Paget’s disease of bone or malignancy, except nonmelanoma skin cancers, carcinoma in situ of the cervix, ductal carcinoma in situ of breast at baseline or through 30 days post-index date, were excluded. No subject’s identity or medical records were disclosed for the purposes of this study. The study only used data de-identified in compliance with 45 CFR 164.514(a)-(c) and therefore all data were accessed using HIPAA compliant procedures.

### Fracture definition

Fractures, including pathologic ones, were considered case qualifying if they were identified during either an inpatient hospitalization (any position on a hospital claim) or an outpatient visit with a repair procedure code, based on a primary or secondary *International Classification of Diseases*, *Ninth Revision* or *Tenth Revision* (ICD-9 or 10) code, listed on the same claim and based on a validated algorithm shown to have accuracy with a positive predictive value exceeding 90% [10]. Fracture episodes that started ˃30 days after the index date at a different anatomic site or those that started after 90 days with no fracture claims at the same anatomic site were considered subsequent fractures.

### Candidate predictor variables

Predictor variables considered during the baseline (12 months pre-index) period included demographic characteristics (ie, age, gender, geography), setting where the fracture occurred (inpatient/outpatient), clinical characteristics (ie, fracture history, site of index fracture, fall history, mobility issues, Parkinson’s disease, stroke), concomitant medications associated with increased fall or fracture risk (ie, muscle relaxants, anxiolytics, sedatives, sleep medications, diuretics), or diseases or medications associated with poor bone quality or healing (ie, liver disease, diabetes, oral corticosteroids) [11]. Comorbid conditions were identified by the presence of a diagnostic claim in an inpatient or outpatient setting prior to index fracture using ICD-9 codes before September 30, 2015 and ICD-10 codes after this date.

Medication use was assessed by the presence of ≥1 prescription claims. Comorbidity scores were calculated per the Quan-Charlson Comorbidity Index using diagnosis codes in the pre-index period and categorized as 0, 1 to 2, 3 to 4, and ≥5 [12].

### Model development and internal validation (ORD)

Logistic regression models were created with binary outcome of a second fracture versus no second fracture within a year of index fracture, with the goal of predicting the occurrence of a second fracture. The model selection process included an examination of several models, which considered all covariates as main effects only as well as main effects and two-way interactions. A stratified analysis was conducted separately for commercial and Medicare Advantage enrollees. Stepwise, forward, and backward selection including/excluding select variables based on clinical and statistical significance were carried out. Performance of each model was subsequently evaluated using the concordance index (c-statistic), and the final set of covariates was chosen to balance parsimony, performance, and interpretation.

Internal validation was conducted using bootstrapping methodology [13] where, for each of 100 bootstrapped samples drawn with replacement: (a) Fit logistic regression model with same set of covariates as from the original model and calculate the c-statistic; (b) Score original dataset using model from (a) and calculate the c-statistic; (c) Difference in c-statistics between (a) and (b) is called the optimism. The optimism is then averaged across all 100 samples, that average is subtracted from the original c-statistic from the observed model and data, and the difference is reported as the internally validated c-statistic.

### Model scoring

**Fig 1:**
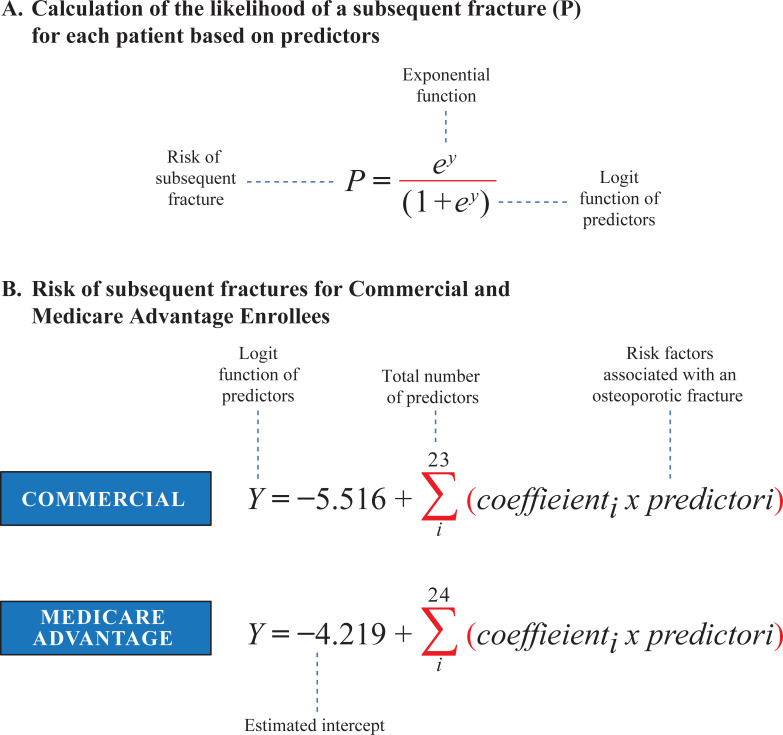
Model scoring. *y* equals the intercept plus the total sum of all the estimates for the predictors associated with the risk of a subsequent fracture. There are 23 predictors of interest in the Commercial and 24 predictors in the Medicare Advantage Enrollees.

The model calculates a patient’s individual probability or risk of a second fracture in the year following the index fracture (prognostic prediction model) using coefficients for the variables relevant to a particular patient. The total score is based on the summation of values corresponding to each predictor variable (Fig 1A) with separate models by insurance type (Fig 1B). The summed value is then converted to a probability. The predicted probability is a patient-level measure. The predicted probabilities should be used with caution as they are dependent on the prevalence of refracture in the population and might be best used as relative or comparisons measures (ie, Patient A is higher risk than Patient B). A threshold for intervention may be set by the end user as to what may be considered a high-risk probability with the objective for identification and treatment of individuals thought to be at imminent risk of fracture.

### External model validation (Symphony data)

Patient cohorts were created using the validated fracture algorithm as described previously. Variables previously thought to be predictive of future fracture risk were considered. Subsequently, the predictive power of the two models and individual variables within them were compared. After validation was completed, additional conditions and concomitant medications, not included in the Optum model, but which were recommended for risk-based assessment by the recently-issued clinical practice guidelines, were considered to further test the fit of the model (S1 Table) [10]. The guidelines suggest an evaluation of factors beyond bone health assessment including comorbidities that increase the risk of falls and/or fractures consistent with recently issued consensus statement regarding secondary fracture prevention [14]. TRIPOD guidelines were followed in reporting of these results [15].

## Results

In the ORD, 197,104 patients were identified with 1) a case-qualifying fracture between January 1, 2007 and May 31, 2017, and 2) ≥12 months of follow-up after the case-qualifying fracture. Forty-three percent (*n* = 84,866) were commercial and 57% (*n* = 112,238) were Medicare Advantage enrollees. Using Symphony data, 1,852,818 met the inclusion/exclusion criteria (Table 1).

**Table 1:** Patient attrition and overall refracture rate in symphony database.

Criteria	*n*	%^a^
Diagnosis for fracture occurring between Sep 1, 2012 and Oct 31, 2018^b^	14,322,416	100%
Case-qualifying eligible fracture^c^	7,676,794	53.6%
Complete demographic information	7,676,794d	100%
Age ≥50 years	4,377,553	57.0%
Limit to patients with Commercial or Medicare Plan	3,004,992	68.6%
No evidence of a diagnosis for Paget’s disease in the baseline through 30 days post-index date	3,002,744	99.9%
No evidence of a diagnosis for cancer (except for nonmelanoma skin cancers, carcinoma in situ of the cervix, ductal carcinoma in situ of breast) at baseline through 30 days post-index date	2,607,623	86.8%
Pre-Enrollment- 1 activity^d^ in months 9–14 of look back	2,174,011	83.4%
Post Enrollment- 1 activity^d^ in months 9–14 of look forward and Pre-Enrollment- 1 activity^d^ in months 9–14 of look back	1,852,818	85.2%
Patients with 2nd fracture occurring within 1 year of index fracture	193,883	10.5%

^a^Percentages remaining from the rows above.

^b^Identification period is based on data availability and the time period is different from that in the Optum study (2007–2017).

^c^Using the validated algorithm as Optum Research Database (10) including an inpatient claim or an outpatient claim along with a procedure repair code or a vertebral fracture with a claim for imaging within 30 days.

^d^Medical or pharmacy claim.

### Population differences

Differences in study populations were expected given the variations in database structures, availability of variables within the databases, and variations in patient populations covered. To assess the comparability of patients in ORD versus Symphony data, we initially evaluated medical and treatment history between the populations (Table 2). Average patient age and percentage of female patients were comparable. With the exception of history of falls (41.26% vs 18.74%) and opioid use (62.80% vs 46.78%), both of which were higher in the ORD, the two populations were mostly comparable.

**Table 2:** Comparison of comorbidity rates and concomitant medications.

Comorbidities that increase fall risk	ORD (N = 197,104)^a^	Symphony (N = 1,852,818)^b^	Absolute Difference
Prior history of stroke	11,742 (5.96%)	126,255 (6.81%)	−0.85%
** History of falls**	**81,325 (41.26%)**	**347,139 (18.74%)**	**22.52%**
Mobility impairments	51,390 (26.07%)	517,644 (27.94%)	−1.87%
Vision impairments	13,201 (6.70%)	138,708 (7.49%)	−0.79%
Parkinson’s disease	3,701 (1.88%)	33,145 (1.79%)	0.09%
Muscle atrophy/muscle weakness/sarcopenia	16,657 (8.45%)	188,542 (10.18%)	−1.73%
**Medications that increase fall risk**			
Alpha blockers	9,067 (4.60%)	110,489 (5.96%)	−1.36%
Anticholinergic antihistamines	8,422 (4.27%)	145,755 (7.87%)	−3.60%
Antipsychotics	1,376 (0.70%)	12,872 (.69%)	0.01%
Barbiturates	308 (0.16%)	1,189 (.06%)	0.10%
Benzodiazepines	30,726 (15.59%)	260,047 (14.04%)	1.55%
Beta blockers	46,392 (23.54%)	403,697 (21.79%)	1.75%
Muscle relaxants	25,243 (12.81%)	307,017 (16.57%)	−3.76%
Nonbenzodiazepine, benzodiazepine receptor agonist	14,695 (7.46%)	136,618 (7.37%)	0.09%
** Opioids**	**123,782 (62.80%)**	**866,661 (46.78%)**	**16.02%**
Proton pump inhibitors	43,568 (22.10%)	474,068 (25.59%)	−3.81%
Selective serotonin reuptake inhibitor	39,506 (20.04%)	480,072 (25.91%)	−5.87%
Tricyclic antidepressant	5,972 (3.03%)	32,668 (1.76%)	1.27%
Vasodilators	8,424 (4.27%)	115,319 (6.22%)	−1.95%
Oral corticosteroids	23,012 (11.68%)	293,538 (15.84%)	−4.16%
**Comorbidities that lengthen healing**			
Diabetes	49,444 (25.09%)	501,141 (27.05%)	−1.96%
Renal disease	29,968 (15.20%)	324,640 (17.52%)	−2.32%
Liver disease	12,990 (6.59%)	190,755 (10.3%)	−3.71%
**Other comorbidities**			
Rheumatoid arthritis	7,360 (3.73%)	76,252 (4.12%)	−0.39%
Hypertension	134,021(68.00%)	1,175,998 (63.47%)	4.53%
Arthritis	71,693 (36.37%)	706,645 (38.14%)	−1.77%
Respiratory diseases	77,524 (39.33%)	797,214 (43.03%)	−3.97%
Alzheimer’s disease	7,851 (3.98%)	45,879 (2.48%)	1.50%
Dementia	24,251 (12.30%)	186,154 (10.05%)	2.25%
Lung disease (COPD, asthma)	41,556 (21.08%)	441,133 (23.81%)	−2.73%
Depression	38,647 (19.61%)	408,484 (22.05%)	−2.44%
Anxiety	27,803 (14.11%)	346,039 (18.68%)	−4.57%
Sleep disorders	26,472 (13.43%)	345,285 (18.64%)	−5.21%
Cardiovascular diseases	153,715(77.99%)	1,383,008 (74.64%)	3.35%
Hypothyroidism	43,232 (21.93%)	384,394 (20.75%)	1.18%
Obesity	18,255 (9.26%)	277,866 (15.00%)	−5.74%

COPD, chronic obstructive pulmonary disease; ORD, Optum Research Database.

^a^43% were commercial and 57% were Medicare Advantage enrollees.

^b^51% were commercial and 49% were Medicare Advantage enrollees.

The most prevalent conditions associated with increased fall risk [11] for both populations were history of falls and mobility impairments. The most prevalent conditions associated with increased length of fracture healing were diabetes and renal disease. The most prevalent chronic conditions included cardiovascular disease (CVD), hypertension, respiratory disease, and arthritis. Commonly prescribed medications associated with increased fall risk [11] included opioids, beta blockers, proton pump inhibitors, and selective serotonin reuptake inhibitors.

### Predictive model results: ORD versus Symphony

Various selection techniques generally selected the same set of variables and therefore had similar predictive performance. Predictive performance of the model, coefficients of all variables included in the model, and a summary of the key differences between the results of the ORD and Symphony model are provided (Tables 3 and 4).

**Table 3:** Comparison of parameter estimates and *P* values from the ORD and Symphony commercial model.

	ORD model		Symphony model	
Parameter	Estimate	*P* Value	Estimate	*P* Value
Intercept	−5.516	< .001	−3.051	< .001
Age	0.024	< .001	0.004	< .001
Male	−0.228	< .001	−0.123	< .001
**Ankle fracture**	**−0.291**	**< .001**	0.050	.001
Carpal fracture	−0.033	.807	−0.069	.001
Hip fracture	0.299	< .001	0.374	< .001
Femur fracture	0.295	< .001	0.291	< .001
Pelvis fracture	0.374	< .001	0.268	< .001
Radius fracture	−0.172	.008	−0.058	< .001
Shoulder fracture	0.140	.037	0.105	< .001
Spine fracture	0.983	< .001	0.557	< .001
Tibia fracture	0.235	.003	0.124	< .001
Stroke	0.238	< .001	0.063	< .001
Mobility issues	0.281	< .001	0.240	< .001
Liver disease	0.276	< .001	0.126	< .001
Respiratory disease	0.204	< .001	0.116	< .001
Depression	0.183	< .001	0.171	< .001
Cardiovascular disease	0.245	< .001	0.198	< .001
Lung disease (COPD, asthma)	0.220	< .001	N/A	N/A
Benzodiazepines	0.177	< .001	0.080	< .001
Nonbenzodiazepines	0.210	< .001	0.011	.388
Selective serotonin reuptake inhibitor (SSRI)	0.159	< .001	N/A	N/A
OP treatment	0.256	< .001	0.169	< .001

COPD, chronic obstructive pulmonary disease; OP, osteoporosis; ORD, Optum Research Database.

### Model validation

After forcing the same variables from the ORD model using Symphony data, the direction and parameter estimates remained consistent with a few notable exceptions. In both the Commercial and Medicare models, the estimate for history of ankle fracture changed from a negative to a positive predictor of subsequent fractures (Tables 3 and 4). In the Medicare model, prior oral corticosteroid use also changed from positive in the ORD model to negative in the Symphony model, though both of these variables were statistically insignificant (Table 4).

**Table 4:** Comparison of parameter estimates and P values from ORD and Symphony Medicare Advantage model.

	ORD model		Symphony model	
Parameter	Estimate	*P* Value	Estimate	*P* Value
Intercept	−4.219	< .001	−2.631	< .001
Age	0.015	< .001	0.001	< .001
Male	−0.250	< .001	-0.113	< .001
OP diagnosis	0.258	< .001	0.223	< .001
Ankle fracture	−0.315	< .001	0.006	.644
Carpal fracture	−0.017	.855	−0.139	< .001
Hip fracture	0.181	< .001	0.239	< .001
Femur fracture	0.074	.111	0.269	< .001
Pelvis fracture	0.289	< .001	0.215	< .001
Radius fracture	−0.129	.005	−0.108	< .001
Shoulder fracture	0.056	.226	0.026	.046
Spine fracture	0.558	< .001	0.447	< .001
Tibia fracture	0.113	.072	0.089	< .001
History of falls	0.085	< .001	0.197	< .001
Mobility issues	0.204	< .001	0.189	< .001
Respiratory disease	0.108	< .001	0.101	< .001
Depression	0.152	< .001	0.135	< .001
Anxiety	0.138	< .001	N/A	N/A
COPD	0.109	< .001	0.100	< .001
Liver disease	0.239	< .001	0.121	< .001
Muscle relaxants	0.191	< .001	0.039	< .001
Nonbenzodiazepines	0.166	< .001	0.019	.129
Selective serotonin reuptake inhibitor (SSRI)	0.116	< .001	N/A	N/A
BMD test	−0.150	< .001	N/A	N/A
**Oral corticosteroids**	**0.118**	**< .001**	**-0.001**	**.890**

BMD, bone mineral density; COPD, chronic obstructive pulmonary disease; OP, osteoporosis; ORD, Optum Research Database.

Where the two models differed were in the magnitude of estimates. In both the Commercial and Medicare models, the Symphony model assigned a higher intercept, but the variable estimates tended to be of lower absolute magnitude (Tables 3 and 4). The fit of the models was comparable (C-statistics for the commercial model: Optum 0.737, Symphony 0.620; C-statistics for the Medicare model: Optum 0.637, Symphony 0.603). After separating commercial and Medicare patients, all models resulted in approximately the same c-statistics suggesting that the models were not overly affected by including or excluding any single variable. Given that the predictors of future fracture risk remained consistent, the direction of the association persisted, and that the fit of the two models were comparable, we consider the ORD model to have been validated using a different database.

### Additional covariates of interest

All of the additional variables were available in the Symphony data, though some had relatively low prevalence (S1 Table in the Supplemental Materials). Most variables were significant predictors of future fracture risk except for acromegaly, Cushing disease, immobile paralysis, seizure disorders, and osteomalacia; however, the fit of the model did not improve in consideration of these characteristics and thus they were not considered. As an additional attempt to improve model accuracy, variables were checked for correlation; pairs with a correlation of 0.3 or higher had an interaction term added. The new variables and interaction terms were introduced to the model and tested for significance and impact with a forward stepwise methodology. The introduction of these variables slightly improved the fit of the models, increasing the c-score in the Commercial and Medicare models from 0.62 to 0.63 and from 0.603 to 0.609, respectively. The increase in predictive power indicated that these variables were not poor inclusions, but it was not representative of a large enough difference to render the original OptumRx model obsolete.

The estimated probability of fracture varied by patient, depending on demographic characteristics and presence or absence of comorbidities and conditions that increased the risk of falls and fractures. For example, in the commercial health plan population, a 59-year-old male with a hip fracture and no comorbidities had a lower risk of fracture (1.75%) than a 70-year-old female with a hip and shoulder fracture who also had a history of CVD (6.7%). In the Medicare Advantage population, a 70-year-old male with a hip fracture and no comorbidities had a lower risk of fracture in the year following his index fracture (3.78%) than a female counterpart with a spine fracture and who was being treated for anxiety (SSRI) and use of oral corticosteroids (OCS), and had a history of falls (19.53%).

## Discussion

The current study focused on the development and validation of a secondary prediction model for fracture. The output is the estimation of the probability of fracture risk in the year following an initial fracture and in a patient with a given number of characteristics over another patient with a different set of characteristics. The C-statistics of 0.737 for the ORD commercial model and 0.637 for the ORD Medicare model indicated 74% or 64% probability of predicting higher second fracture risk for a randomly selected actual second fracture patient than a randomly selected non-second fracture patient. Both model-based predictions are better than chance (0.5). The performance of the developed and internally validated ORD model was tested and externally validated in a new population of patients. The validation of the model is indicative of its robustness.

In addition to having a good performance in the development sample, the model performed well in a different population of patients supporting its generalizability. Specifically, some differences in the population characteristics in the ORD and Symphony models, including a higher history of falls and opioid use in ORD, are important given their association with increased fracture risk. We believe that the higher opioid use is due to fewer restrictions for use in the time period of the ORD model compared to today. A history of falls, a known risk factor for fractures, continues to play a role in secondary fracture risk and remains an important consideration for risk assessment.

The use of OCS was a positive predictor in ORD and a negative predictor in Symphony Medicare patients. Overall, higher steroid use was noted in Symphony; however, this was not limited to chronic steroid users. While we were able to adjust for certain conditions typically associated with steroid use (ie, arthritis, COPD), we did not have detailed clinical information on other factors associated with pre-treatment bone density (ie, weight) that could further impact the effect of steroids on fractures.

In addition to mobility issues, other concomitant medications [16] used to treat patient comorbidities need evaluation and consideration in fracture risk assessment. Both commercial and Medicare models had respiratory disease, depression, and liver disease as risk factors for secondary fractures, whereas stroke, mobility issues, and CVD were also predictors of risk in the commercial population. While mobility issues increase fall risk and subsequent fractures directly, CVD and stroke may be associated with increased fracture risk due to shared etiology or risk factors or as a marker of frailty [17].

Our model has several advantages over the FRAX—a risk assessment tool used to evaluate the 10-year probability of hip and major osteoporotic fractures [18,19]. First, our model predicts one-year risk for patients who have already incurred a fragility fracture, thus focusing on secondary fracture prevention. Second, our model predicts all fractures not just hip and major osteoporotic fractures. Third, our model is transparent providing the model parameters. The model recognizes treatment and treatment failure could occur and includes consideration of osteoporosis treatment received [20]. In comparison, FRAX is to be used in treatment-naïve patients. Finally, our model included population characteristics that are readily available from the patient’s health records (ie, comorbidities and concomitant medications associated with increased fall and fracture risk) and not subject to reporting bias associated with self-reporting of lifestyle risk factors required by FRAX. Our model is therefore more closely related to a population risk management tool because the output of the query is a list of patients at risk for subsequent fracture.

The model is also flexible and predicts outcomes for two different patient populations (commercial and Medicare enrollees). Each provider or health system may have a large number of patients in one or the other category and may want to run the model separately for individuals in the population of interest. The model does not require a special software tool. Microsoft Excel or IBM’s Statistical Package for the Social Sciences can be used for calculations using the coefficient estimates and a list of comorbidities, concomitant medications, and osteoporosis disease and treatment history.

The model has several limitations, mainly due to the retrospective nature and inherent features of an administrative claims database. First, the database, like other claims data, is structured largely to collect information for billing purposes and not research. Any errors or inconsistencies in the documentation of diagnosis or medication codes may lead to misclassification of patients; however, we expect such errors to be low because the proper documentation of information in claims is a prerequisite for reimbursement. The ORD allows for the identification of individuals with guaranteed coverage during the desired period, while Symphony data does not. To maximize the opportunity to include the full spectrum of claims for patients in Symphony, patients were required to have recorded activity in the 9–14 months pre- and post-index fracture, which provided sufficient certainty that the patient remained in the database during the time periods. Second, the model is US-centric and not validated in other regions/countries, whereas other models, such as FRAX, provide calculations separately for different regions/countries based on local epidemiology and population characteristics. Because we used administrative data, we could not consider environmental factors that increase fall risk in the patient’s residence [11]. Lastly, our data did not specifically evaluate future fracture risk for institutionalized patients as data were not available; however, we did include conditions that are prevalent in this population including diseases associated with poor mobility and balance.

Fractures are associated with increased disease burden even years after fracture incidence [21]. In an evaluation of longer term outcomes of women 70 years of age, the mean utility decrement due to fractures was 12-fold greater in patients with sentinel hip fracture and was increased 15-fold for spinal, 4-fold for forearm, and 8-fold for humeral fracture, highlighting the importance of secondary fracture prevention [8]. These data further emphasize the importance of secondary fracture prevention especially given the low rate of treatment initiation and adherence following fracture incidence. Information on risk of subsequent fracture may guide healthcare professionals in their decision-making regarding testing or initiation of treatment. The information could potentially modify patient attitude and behavior regarding their risk and subsequently impact treatment adherence.

In summary, the model suggests consideration of factors beyond bone density, including comorbidities and concomitant medications associated with an increased risk of falls, fractures, or reduced bone health/quality. Future research includes implementation in a health system to determine the usability in the fracture care pathway and impact on patient outcome.

## Supporting information

S1 Checklist TRIPOD checklist: Prediction model development and validation. (PDF)

S1 Table Rates for added comorbidities (based on AACE/ACE 2020 guidelines). (DOCX)

## References

[1] Lewiecki EM, Wright NC, Curtis JR, Siris E, Gagel RF, Saag KG, et al. Hip fracture trends in the United States, 2002 to 2015. Osteoporos Int. 2018;29(3):717–722. doi: 10.1007/s00198-017-4345-0 29282482

[2] Lewiecki EM, Chastek B, Sundquist K, Williams SA, Weiss RJ, Wang Y, et al. Osteoporotic fracture trends in a population of US managed care enrollees from 2007 to 2017. Osteoporos Int. 2020;31(7):1299–1304. doi: 10.1007/s00198-020-05334-y 32062687 PMC7280339

[3] Balasubramanian A, Tosi LL, Lane JM, Dirschl DR, Ho P-R, O’Malley CD. Declining rates of osteoporosis management following fragility fractures in the U.S., 2000 through 2009. J Bone Joint Surg Am. 2014;96(7):e52. doi: 10.2106/JBJS.L.01781 24695929

[4] CMS Centers for Medicare & Medicaid Services. Medicare 2018 Part C & D Star Ratings Technical Notes. Available from: https://www.cms.gov/Medicare/Prescription-Drug-Coverage/PrescriptionDrugCovGenIn/Downloads/2018-Star-Ratings-Technical-Notes-2017_09_06.pdf. Accessed November 19, 2020.

[5] Song X, Shi N, Badamgarav E, Kallich J, Varker H, Lenhart G, et al. Cost burden of second fracture in the US health system. Bone. 2011;48(4):828–836. doi: 10.1016/j.bone.2010.12.021 21211578

[6] Weaver J, Sajjan S, Lewiecki EM, Harris ST, Marvos P. Prevalence and cost of subsequent fractures among U.S. patients with an incident fracture. J Manag Care Spec Pharm. 2017;23(4):461–471. doi: 10.18553/jmcp.2017.23.4.461 28345441 PMC10398116

[7] Williams SA, Chastek B, Sundquist K, Barrera-Sierra S, Leader D Jr, Weiss RJ, et al. Economic Burden of Osteoporotic Fractures in US Managed Care Enrollees. Am J Manag Care. 2020;26(5):e142–e149. doi: 10.37765/ajmc.2020.43156 32436682

[8] Kanis JA, Johansson H, Odén A, Harvey NC, Gudnason V, Sanders KM, et al. Characteristics of Recurrent fractures. Osteoporos Int. 2018;29(8):1747–1757. doi: 10.1007/s00198-018-4502-0 29947869 PMC6076437

[9] Rubin KH, Abrahamsen B, Friis-Holmberg T, Hjelmborg JV, Bech M, Hermann AP, et al. Comparison of different screening tools (FRAX®, OST, ORAI, OSIRIS, SCORE and age alone) to identify women with increased risk of fracture. A population-based prospective study. Bone. 2013;56(1):16–22. doi: 10.1016/j.bone.2013.05.002 23669650

[10] Wright NC, Daigle SG, Melton ME, Delzell ES, Balasubramanian A, Curtis JR. The design and validation of a new algorithm to identify incident fractures in administrative claims data. J Bone Miner Res. 2019;34(10):1798–1807. doi: 10.1002/jbmr.3807 31170317

[11] Camacho PM, Petak SM, Binkley N, Diab DL, Eldeiry LS, Farooki A, et al. American Association of Clinical Endocrinologists/American College of Endocrinology Clinical Practice Guidelines for the Diagnosis and Treatment of Postmenopausal Osteoporosis-2020 Update. Endocr Pract. 2020;26(Suppl 1):1–46. doi: 10.4158/GL-2020-0524SUPPL 32427503

[12] Quan H, Li B, Couris CM, Fushimi K, Graham P, Hider P, et al. Updating and validating the Charlson comorbidity index and score for risk adjustment in hospital discharge abstracts using data from 6 countries. Am J Epidemiol. 2011;173(6):676–682. doi: 10.1093/aje/kwq433 21330339

[13] Steyerberg EW, Harrell FE Jr, Borsboom GJ, Eijkemans MJ, Vergouwe Y, Habbema JD. Internal validation of predictive models: efficiency of some procedures for logistic regression analysis. J Clin Epidemiol. 2001;54(8):774–781. doi: 10.1016/s0895-4356(01)00341-9 11470385

[14] Conley RB, Adib G, Adler RA, Åkesson KE, Alexander IM, Amenta KC, et al. Secondary Fracture Prevention: Consensus Clinical Recommendations from a Multistakeholder Coalition. J Bone Miner Res. 2020 Jan;35(1):36–52. doi: 10.1002/jbmr.3877 31538675

[15] Patzer RE, Kaji AH, and Fong Y. TRIPOD reporting guidelines for diagnostic and prognostic studies. JAMA Surg. 2021 Published online April 07, 2021. doi: 10.1001/jamasurg.2021.0537 33825807

[16] Pharmacist’s Letter/Prescriber’s Letter. Potentially Harmful Drugs in the Elderly: Beers List. June 2012. Available from: http://medischebijsluiter.nl/wp-content/uploads/2017/02/Beers-Criteria-Literature.pdf. Accessed November 19, 2020.

[17] Samelson EJ, Kiel DP, Broe KE, Zhang Y, Cupples LA, Hannan MT, et al. Metacarpal Cortical Area and Risk of Coronary Heart Disease: The Framingham Study. Am J Epidemiol. 2004;159(6):589–595. doi: 10.1093/aje/kwh080 15003963

[18] Centre for Metabolic Bone Diseases. FRAX Fracture Risk Assessment Tool. Available from: https://www.sheffield.ac.uk/FRAX/. Accessed November 19, 2020.

[19] Kanis JA, Johnell O, Oden A, Johansson H, McCloskey E. FRAX and the assessment of fracture probability in men and women from the UK. Osteoporos Int. 2008;19(4):385–397. doi: 10.1007/s00198-007-0543-5 18292978 PMC2267485

[20] Imel EA, Eckert G, Modi A, Li Z, Martin J, de Papp A, et al. Proportion of osteoporotic women remaining at risk for fracture despite adherence to oral bisphosphonates. Bone. 2016;83:267–275. doi: 10.1016/j.bone.2015.11.021 26657827

[21] Gold T, Williams SA, Weiss RJ, Wang Y, Watkins C, Carroll J, et al. Impact of fractures on quality of life in patients with osteoporosis: a US cross-sectional survey. J Drug Assess. 2019;8(1):175–183. doi: 10.1080/21556660.2019.1677674 31692954 PMC6818103

