# Supplementary material for: Development and validation of a one year predictive model for secondary fractures in osteoporosis
Source: PLoS One. 2021 Sep 27;16(9):e0257246. doi: 10.1371/journal.pone.0257246 (PMC8475984; doi:10.1371/journal.pone.0257246)
Supplement: S1 Table — (DOCX) [file pone.0257246.s002.docx]

**S1 Table. Rates for added comorbidities (based on AACE/ACE 2020 Guidelines).**

| **Comorbidity, n (%)** | ***N* = 1,852,818** |
| --- | --- |
| Hyperparathyroidism | 14,999 (0.81%) |
| Acromegaly | 0 (0.0%) |
| Cushing disease | 1,438 (0.08%) |
| Vitamin D deficiency | 152 (0.01%) |
| Hypogonadal males | 27,186 (1.47%) |
| Eating disorder | 1,615 (0.09%) |
| Gastric bypass surgery | 27,070 (1.46%) |
| Osteomalacia | 2,771 (0.15%) |
| Immobile paralysis | 2,474 (0.13%) |
| Renal transplant | 5,146 (0.28%) |
| Liver transplant | 1,888 (0.1%) |
| Heart transplant | 808 (0.04%) |
| Lung transplant | 675 (0.04%) |
| Pancreas transplant | 527 (0.03%) |
| Family history of osteoporotic fracture | 3,634 (0.2%) |
| Low body mass | 4,538 (0.24%) |
| Tobacco use | 334,078 (18.03%) |
| Alcohol abuse | 39,429 (2.13%) |
| Thoracic kyphosis | 10,519 (0.57%) |
| Impaired hearing | 119,361 (6.44%) |
| Seizure disorders | 80,283 (4.33%) |
| Autonomic dysfunction with orthostatic hypotension | 814,557 (43.96%) |
